# Editorial: Neural circuits and neuroendocrine mechanisms of major depressive disorder and premenstrual dysphoric disorder: Toward precise targets for translational medicine and drug development

**DOI:** 10.3389/fpsyt.2022.983604

**Published:** 2022-07-26

**Authors:** Sheng Wei, Fushun Wang, Jianfeng Liu, Yang Wang

**Affiliations:** ^1^Experimental Center, Shandong University of Traditional Chinese Medicine, Jinan, China; ^2^Institute of Brain and Psychological Science, Sichuan Normal University, Chengdu, China; ^3^Department of Psychological and Brain Sciences, College of Liberal Arts, Texas A&M University, College Station, TX, United States; ^4^Department of Integrative Medicine, Xiangya Hospital of Central South, Changsha, China

**Keywords:** major depressive disorder, premenstrual dysphoric disorder, neural circuits, neuroendocrine, translational medicine

With the intensified social competition and increased psychological pressure in modern society, the impact of emotions on people' health has attracted increasing attention from around the world. Major depressive disorder (MDD) and premenstrual dysphoric disorder (PMDD) are the two common types of depressive disorders described in The Diagnostic and Statistical Manual of Mental Disorders (DSM-5) (1). Depressive disorder, represented by PMDD and MDD, is the most common mental illness in modern society, and has a chronic and recurrent course (2). With the development of social economy and the influence of factors such as unemployment as well as changes in life rhythm, depressive disorder has become one of the most serious diseases threatening human health. According to a survey conducted by the World Psychiatric Association, the incidence rate of depressive disorder worldwide is currently 4.2%, while that in China is 6.9%, with an annual growth rate of 113%. According to data from the Global Burden of Disease Organization, mental/neurological diseases occupy the number-one spot in terms of the burden of disease, of which depressive disorder is first among mental/neurological diseases (3).

Although the pathogenesis of depressive disorder has not yet been fully elucidated, there are several clues that neural circuits and neuroendocrine pathways are involved in pathogenesis and drug intervention mechanisms (4–7). Substantial evidence exists for significant alterations in the interactions of relevant brain regions and their neural circuits in MDD (6, 7). Functional Magnetic Resonance Imaging (fMRI) methods have been widely used in clinical research on the mechanism of MDD neural circuits, most of which focus on related brain regions involved in emotion regulation and cognitive function, such as the amygdala, cingulate cortex, prefrontal cortex, striatum, and insular cortex (8, 9). In terms of animal experiments, the synaptic plasticity (such as synaptic density and synaptic protein density) in the prefrontal cortex, nucleus accumbens, amygdala, and hippocampus of MDD model animals was significantly altered (10, 11), while the incidence of PMDD is more closely related to neuroendocrine factors. The premenstrual period is a period of rapid fluctuations in the levels of female reproductive hormones. During the premenstrual phase, the concentration of estrogens, such as estradiol and progesterone, in the body increases significantly, reaching its highest level in the middle and late luteal phase. Get used to it gradually. After menstruation, estrogens show a rapid “withdrawal” phenomenon, returning to the normal range. Some researchers have proposed that this “withdrawal” phenomenon is the most critical reason for premenstrual dysphoric disorder, an idea that has been summarized as the “ovarian-steroid-withdrawal hypothesis” (12, 13). Animal experiments have shown that rapid withdrawal of progesterone can induce PMDD-like behavior in animals, an effect that can also be mimicked by blocking progesterone metabolism (reducing allopregnanolonein [ALLO] levels) (14, 15). As a positive allosteric regulator of γ-aminobutyric acid receptors (GABARs), the sudden drop in ALLO after menstruation leads to abnormal regulation of the function of GABARs, which plays an important role in the pathogenesis of PMDD (16, 17). In clinical terms, the risk of PMDD can be reduced by supplementing ALLO levels, which can be achieved *via* certain antidepressant treatments (18, 19).

The regulation of emotions by the brain is specific to certain brain regions and cells and involves complex changes in neural circuits and neuroendocrine levels. However, the lack of clarity regarding the drug target and mechanism greatly limit translational medicine and drug development. The current treatment drugs are mainly selective serotonin reuptake inhibitors (SSRIs). Overall, 30–40% of patients with MDD or PMDD are insensitive to drug treatment and experience substantial psychiatric side effects and slow onset of action. This can also produce drug resistance, with obvious time lag and inefficiency (20). Therefore, further research into the neural circuits and neural endocrine mechanisms of MDD and PMDD as well as treatment moving toward translational medicine and drug development are keys to solving the above problems. Meta-analysis and animal experimental evidence have shown the high potential of complementary and alternative therapies in the treatment of PMDD and MDD (21, 22). Unlike single-target chemical drugs, Chinese medicine therapy can regulate specific neural circuits and neuroendocrine functions with multiple targets and pathways to treat these diseases, avoiding the side effects of chemotherapy and representing a promising therapeutic direction.

In consideration of the aforementioned realization, we organized this special issue to advance our understanding of the pathogenesis of MDD and PMDD, particularly regarding neural circuits and neuroendocrine mechanisms. This information will provide a basis and possible clues for clinical treatments and drug development. For this Research Topic, we invited recent studies that focus on the neural circuits and neuroendocrine mechanisms of PMDD and MDD and received 11 submissions. After a half year of critical peer review, nine papers have been accepted.

In the experimental report titled “*Decreased Plasma Hydrogen Sulfide Level Is Associated With the Severity of Depression in Patients With Depressive Disorder*,” Yang et al. recruited 47 depressed patients and 51 healthy individuals and found that decreased H_2_S is involved in the pathophysiology of depression as well as that plasma H_2_S may be a potential indicator for depression severity.

In the paper titled “*Antidepressant Treatment-Induced State-Dependent Reconfiguration of Emotion Regulation Networks in Major Depressive Disorder*,” Zhao et al. collected data from 70 MDD patients and 43 sex- and age-matched healthy controls and found that four dFC states were identified in the emotion networks. Their alterations of state-related occurrence proportion were found in MDD and subsequently normalized following 12-week antidepressant treatment. Baseline strong dFC predicted the reduction rate of Hamilton Depression Rating Scale (HAMD) scores.

In Yu et al.'s paper, titled “*Serum Lipid Concentrations Are Associated With Negative Mental Health Outcomes in Healthy Women Aged 35-49 Years*,” the authors recruited 319 healthy participants and found that there was a significant association between K10 scores and metabolic parameters, including Body Mass Index (BMI), total and LDL cholesterol, and triglycerides.

In the paper titled “*Sleep Disturbances and Depression Are Co-morbid Conditions: Insights From Animal Models, Especially Non-human Primate Model*,” Li et al. evaluated the prevalence, clinical features, phenotypic analysis, and pathophysiological brain mechanisms of depression-related sleep disturbances and emphasized the current situation, significance, and insights from animal models of depression.

In Chang et al.'s paper “*Depression Assessment Method: An EEG Emotion Recognition Framework Based on Spatiotemporal Neural Network*,” the authors proposed a novel EEG emotion recognition framework for depression detection, which provides a robust algorithm for real-time clinical depression detection based on EEG.

In the paper titled “Brain Activation during Processing of Depression Emotion in College Students with Premenstrual Syndrome in China: Preliminary Findings,” Gao et al. investigated 13 PMS patients and 15 healthy controls and found that abnormal functional regulation of brain regions such as the occipital lobe and cerebellum leads to abnormal changes in emotional regulation, cognitive ability, and attention distribution in PMS patients, implying significant central pathogenesis.

In the paper “*An End-to-End Depression Recognition Method Based on EEGNet*,” Liu et al. proposed an end-to-end deep learning framework for MDD diagnosis based on EEG signals and found that the method is highly accurate for the diagnosis of MDD and can be used to develop an automatic plug-and-play EEG-based system for diagnosing depression.

In another study titled “*Does Childhood Adversity Lead to Drug Addiction in Adulthood? A Study of Serial Mediators Based on Resilience and Depression*,” He et al. conducted a thorough investigation of the mental status from 937 participants and found that depression led to drug addiction, while resilience weakened the effect of adverse childhood experiences on depression and drug addiction.

In Gu et al.'s paper, “*The Relationship Between 5-Hydroxytryptamine and Its Metabolite Changes With Post-stroke Depression*,” the authors reviewed the relationship of post-stroke depression with three monoamines and emotions. Moreover, they summarized the advantages of psychological therapy in recent years and posted some suggestions for the pharmacology and psychotherapy of post-stroke depression.

Collectively, these studies have thoroughly investigated the neural circuits and neuroendocrine mechanisms of MDD and PMDD as well as some diagnostics and interventions for these emotional diseases. Because neural circuits and neuroendocrine mechanisms are heavily involved in the pathogenesis of MDD and PMDD and the mechanism of drug intervention, multi-faceted exploration in this field will further reveal the underlying neurobiological mechanisms, thereby promoting translational medicine and drug development.

## Author contributions

SW, FW, JL, and YW wrote the paper. All authors contributed to the article and approved the submitted version.

## Funding

The paper was supported by grants from the National Natural Science Foundation of China (Nos. 81974553 and 82004078), the Natural Science Foundation of Shandong Province (Nos. ZR2020ZD17 and ZR2021LZY018), and the Chinese Medicine and Brain Science Youth Scientific Research Innovation Team, Shandong University of Traditional Chinese Medicine (No. 22202101).

## Conflict of interest

The authors declare that the research was conducted in the absence of any commercial or financial relationships that could be construed as a potential conflict of interest.

## Publisher's note

All claims expressed in this article are solely those of the authors and do not necessarily represent those of their affiliated organizations, or those of the publisher, the editors and the reviewers. Any product that may be evaluated in this article, or claim that may be made by its manufacturer, is not guaranteed or endorsed by the publisher.
